# A Treatise on Food and Diet: With Observations on the Dietetical Regimen, Suited for Disordered States of the Digestive Organs; and an Account of the Dietaries of Some of the Principal Metropolitan and Other Establishments for Paupers, Lunatics, Criminals, &c.

**Published:** 1844-02

**Authors:** 


### REVIEWS.

---

Art. V.—*A Treatise on Food and Diet:* with observations on the Dietetical Regimen, suited for *Disordered States of the Digestive Organs;* and an Account of the Dietaries of some of the principal Metropolitan and other Establishments for Paupers, Lunatics, Criminals, Children, the Sick, &c. By Jonathan Pereira, M.D., F.R.S., &. L.S., Licentiate of the Royal College of Physicians in London, &c. &c. Edited by Charles A. Lee, M.D. New York: J. & H. G. Langley. 1vol. 8vo. pp. 325. 1843.

In our last number we noticed, at considerable length, the Elements of Materia Medica, by Pereira—an encyclopedia of knowledge in that department of medical science—by the common consent of the profession, the most elaborate and scientific treatise on Materia Medica in our language. We have now to call the attention of our readers to a work by the same author on a cognate branch of science—the Materia Alimentaria—to a treatise on Food and Diet. They will agree with us, we suppose, that what one eats is a matter not less worthy of grave consideration, than what he takes from his apothecary. The study of the composition of aliments, and their adaptation to the various conditions of the sick, is one in which physicians must feel the liveliest interest. They know that their best directed remedial efforts are often rendered abortive, by the indulgence of their patients in unsuitable articles of diet. Daily it is their experience how difficult it is to impress upon nurses, and the friends of sick people, the importance of seconding their medicines by proper dietetic measures. A treatise, then, embracing the latest discoveries on this subject, and presenting a summary of all the most important facts known in relation to the chemical constitution of alimentary substances, digested by a mind vigorous, and logical in its action, and stored with science, cannot prove otherwise than highly acceptable to the profession. Such a work is Pereira's. The industry, research, and judgment evinced in his earlier production, are the qualities required in the preparation of a treatise on Food and Diet. In reading this work, one is brought up to the present state of the extant knowledge on the subject. Its pages are enriched by the facts brought to light by the labors of Dumas, Liebig, and Boussingault, and the brilliant discoveries made in organic chemistry by these and other chemists, within the last few years, are faithfully reflected in it. A summary of all that chemical analysis has revealed, or experience taught, of the composition and qualities of the various substances which form our aliment, is to be found in this treatise.

We are glad that the work has been issued in so cheap a form, as well as that the style is such as to adapt it to the general reader, which circumstances, it is to be hoped, will insure it a wide circulation, and cause a knowledge of its valuable facts and conclusions to be generally diffused.

The author is burdened with no cherished theory, which he feels bound to sustain on all occasions. Facts from all authorities, chemical or physiological, are fairly and fully stated. He weighs testimony impartially, and is ready to receive the truth from whatever quarter it may come. He rejects the classification of alimentary principles, proposed by his countryman, Prout, and adopts that of the German chemist and physiologist, Tiedemann. In a word, he displays, throughout, an unprejudiced as well as enlarged and philosophical mind, a patience of research, and powers of reasoning which satisfy us, that the preparation of such a work could not have been committed to abler hands.

He adopts, as has just been stated, the classification of Tiedemann, who considers the subject of Food under the heads of *Alimentary Principles*, and *Compound Aliments.* The former uniting compose the several articles of diet. Thus meat consists mainly of fibrine, albumen, gelatine, hæmatosin, fat, and water, alimentary principles; and wheat, of starch, gluten, sugar, and gum, belonging also to that class. But the alimentary principles are themselves compound substances. They contain varying numbers of simple, or undecomposed bodies. Fibrine, for example, consists of carbon, hydrogen, nitrogen, oxygen, phosphorus, and sulphur, known as chemical elements; the study of which is preliminary to the consideration of elementary principles.

Chapter I, therefore, treats of the *Chemical Elements of Foods*, of which there are thirteen; namely, carbon, hydrogen, oxygen, nitrogen, phosphorus, sulphur, iron, chlorine, sodium, calcium, potassium, magnesium, and fluorinē. These, our author alleges, are the essential constituents of the human body, the presence of arsenic, lead, and copper, said to have been occasionally detected by certain chemists, resting, in his judgment, upon insufficient testimony. They are derived from our food, for "a living body," as Dr. Pereira correctly observes, "has no power of forming elements, or converting one elementary substance into another." It is possible that some of the bodies now esteemed elementary, may yet be decomposed and proved to consist of two or more elements. This is the conjecture of Prout, who believes that the lime found in the skeleton of the young chick is obtained by the transmutation of some of the so-called elements, which, under extraordinary circumstances he supposes the vital agents can effect. This, however, does not affect the truth of the proposition, that the living body cannot *create* an element, or change one element into another, for which, probably, no one will now contend.

Proceeding to notice the chemical elements individually, he commences with carbon, one of the most extensively distributed of substances.

"Carbon is an essential constituent of every living or organized tissue, both vegetable and animal. It is, therefore, a necessary ingredient of food," and, accordingly, it is found in the seeds which afford nourishment to the embryo plant, in the yolk of eggs, which supply the young bird with food, and in the milk, "upon which young mammals subsist during the first period of their existence after birth."

The author gives a table showing the quantity of carbon contained in different foods, from which it appears that the nitrogenized substances—the *proteine compounds*—are composed of a little more than 54 per cent. of this element, and that the article of food richest in carbon is hog's lard, into which it enters in the proportion of 79.098 per cent. The quantity of carbon consumed, in the form of food, must, therefore, vary very considerably in different individuals, and in the same individual at different times. Liebig states that an adult, taking moderate exercise, consumes 15.3 oz. avoirdupois of carbon daily, and the statement rests upon observations made with care upon the food consumed by a number of soldiers in barracks. Assuming, then, that each of these soldiers consumed daily about one pound, avoirdupois, of carbon, and that they neither gained nor lost in weight, which is probable, what, asks our author, became of the carbon thus taken in the form of food? As it does not pass off with the excrements, there can be but one answer given to the question, and that is the one which Pereira makes, "that it is thrown out of the system by the lungs and the skin in the form of carbonic acid." He continues:

"By the union of carbon and oxygen, in whatever part of the system this is effected, heat must be evolved. At least, in all other cases, the formation of carbonic acid is attended with the evolution of heat; and we have a right, therefore, to assume, that the same takes place within the body. We are, in fact, acquainted with no conceivable reason why it should be otherwise. Now, according to Despretz, one pound of pure charcoal evolves, by its combustion in oxygen gas, sufficient heat to raise the temperature of 78lbs. of water from 32° Fahr. to 212° Fahr.; and this must be about the amount evolved in the case of the Darmstadt soldiers, independently of the heat produced by the union of oxygen with hydrogen hereafter to be noticed.

"It appears to me that we have a sufficient explanation of animal temperature in the chemical changes just referred to. Indeed, it cannot be doubted that a large proportion, if not the whole, of the heat evolved by animals, is produced by chemical action. But it is scarcely to be expected that experiments can be so nicely and delicately performed as to demonstrate in a precise manner the truth of this chemical theory of animal heat; for while, on the one hand, considerable difficulty is experienced in determining the actual quantity of combustible matter oxidated in the system, it is almost impossible on the other, to estimate, with absolute nicety, the amount of heat actually imparted by a living animal to surrounding bodies. The results of our experiments, therefore, can only furnish, at the most, approximations to the truth.

"Liebig has endeavored to show, that by the conversion of starch or sugar into fat, oxygen is supplied to the system; and that by the union of this disengaged oxygen with carbon (from the bile, for example) heat is developed. Suppose 1 equivalent of carbonic acid, $CO_2$, and 7 equivalents of oxygen, $O_7$, to be abstracted from one equivalent of starch, $C_{12} H_{10} O_{10}$, we have, in the residue, the empirical formula for fat, $C_{11} H_{10} O$.

RELATIVE COMPOSITION OF STARCH AND FAT.

| | | | |
|---|---|---|---|
| 1 eq. Starch . . | $C_{12} H_{10} O_{10}$ | 1 eq. Fat, . . . | $C_{11} H_{10} O$ |
| | | 1 eq. Carbonic Acid C | $O_2$ |
| | | 7 eq. Oxygen . . . | $O_7$ |
| | | | $C_{12}H_{10}O_{10}$ |

"The oxygen thus presumed to be separated from the starch can only be disengaged in the form of either carbonic acid or water, or of both; therefore it must have combined with carbon or hydrogen, or both. Now, Liebig has adduced several reasons for presuming that heat must attend the formation of carbonic acid under these circumstances. 'Thus,' says this distinguished chemist, 'in the formation of fat, the vital force possesses a means of counteracting a deficiency in the supply of oxygen, and consequently in that of the heat indispensable for the vital process.'

"In the natural and healthy condition of the system, the food supplies the necessary carbon for the support of animal heat, but when food is withheld, the fat of the body is consumed; its carbon being converted into carbonic acid, its hydrogen into water. Experience has satisfactorily shown that the heat of the blood in health is the same in all climates and in all conditions of atmospheric temperature. Now it follows that a larger quantity of combustible matter is required in cold climates and cold weather, for keeping up this temperature, than in hot climates and warm weather; since a greater amount of heat must be given off to surrounding media in the former than in the latter. Hence the necessity for a more liberal supply of food in cold weather."

Still, our author is not prepared to go the length of asserting, with Liebig, that if we were to go naked, as the Indians, or if in hunting or fishing we were exposed to the same degree of cold as the Samoyedes, "we should be able to consume the half of a calf, besides a dozen of candles." The influence of a frozen region in augmenting the appetite is admitted, but it is an error, he maintains, "to ascribe the voracity and gormandizing powers of some of the natives of these regions to the influence of cold only." He is rather disposed to look for the strength of the propensity to eat, "in an organ of alimentiveness," the activity of which leads to gluttony in individuals of all nations. He agrees with Liebig, however, that diseases of the liver in tropical climates and hot seasons, may arise from the accumulation of carbon in the system. His words are,

"When the external temperature is high, less carbon is requisite to support the natural heat of the body, and in consequence of the air being expanded, we inhale, at each inspiration, less oxygen by weight than in colder climates and seasons. If, therefore, we continue to consume large quantities of food, there will be an excess of carbonaceous matter in the system.

"The influence of external temperature, excess of food, and want of exercise, on the condition of the liver, is well shown in the goose. The celebrated *pâtés de fois gras*, prepared at Strasburg, are made of the livers of geese, artificially enlarged, 'by the cruel process of shutting the birds up in coops, within a room heated to a very high temperature, and stuffing them constantly with food.'

"In tropical climates and in hot seasons the system requires a smaller quantity, and a less carbonaceous quality, of food than in colder countries and in cold seasons; and the frequent occurrence of hepatic disease among Europeans, who reside in tropical countries, is probably in part owing to their continued employment of a dietetical system fitted for colder climates."

Hydrogen, like carbon is an essential constituent of every organized tissue, and a necessary ingredient in the food of every living thing. It abounds in the farina and oil of seeds, in the albumen of eggs, and in all the constituents of milk. As the carbon is converted into carbonic acid, the hydrogen, in its ultimate changes in the system, must become water, and in the change must evolve much heat. One pound of hydrogen, according to Despretz, by combustion with oxygen, liberates sufficient heat to raise the temperature of 236lbs. of water from 32° to 212°. Hence, remarks Dr. Pereira,

"Part of the heat developed in carnivorous animals must arise from the oxidation of hydrogen; for, in the first place, hydrogen (as of the fat) disappears from the system, and there is no other mode by which it can have done so except by union with oxygen, and its consequent conversion into water. In the second place, of the atmospheric oxygen taken into the lungs during inspiration, the whole is not found, in the inspired air, in union with carbon, nearly every experimenter having detected a loss."

Oxygen is a large and indispensable ingredient of our food. No other element is so abundant in nature, or performs so many important offices. In almost every change which takes place among natural bodies it is an essential agent, and is so intimately connected with the processes of life, that they speedily cease when its influence is withdrawn. As it causes combustion in the lamp by uniting with the oil, and developes electricity in the voltaic battery by oxidizing the metal, "so animal life seems to be inseparably connected with the influence of oxygen on the organism. Interrupt the influence of oxygen, and the flame is extinguished, the electrical current is stopped, and all vital phenomena cease." In each case, the action of the oxygen seems destructive—matter is consumed, or converted into other shapes. The metal is oxidized and dissolved, the oil exhales as carbonic acid, and parts of the animal body also are converted into this gas. Oxygen, then, the *vital* air, acts on the organism to consume it; and "man, and every other animal, are exposed at every period of their lives to the unceasing and destructive action of the atmosphere; with every breath he expires a part of his body, every moment of his life he produces carbonic acid, the carbon of which his food must replace."

The relative proportions of oxygen and carbon in certain kinds of food are given in the following table, taken from Liebig:

**RELATIVE PROPORTIONS OF CARBON AND OXYGEN IN ALIMENTARY PRINCIPLES.**

| | | |
|---|---|---|
| In Fats (on an average) - - - | 120 eq. of Carbon | 10 eq. of Oxygen. |
| In Fibrine, Albumen, and Caseine | 120 " | 36 " |
| In Starch - - - - - - - - - - | 120 " | 100 " |
| In Cane Sugar - - - - - - - - | 120 " | 110 " |
| In Gum . - - - - - - - - - | 120 " | 110 " |
| In Sugar of Milk - - - - - - | 120 " | 120 " |
| In Grape Sugar - - - - - - | 120 " | 140 " |

The carbon and hydrogen of the food being, for the most part, thrown out of the system in combination with oxygen, it follows that the consumption of oxygen must vary with the quality of food, being greater when animal food is eaten, and less when the diet is chiefly composed of vegetables. This view is confirmed by the experience of the diver, Spalding, who consumed more of that air in his diving-bell, when he had used a diet of animal food, and also by the experiments of Fyfe, who found that the consumption of oxygen was greatly reduced by the employment of vegetable diet.

Nitrogen is the next element of the vital tissues. "The chief ingredients of the blood, says Liebig, "contain nearly 17 per cent. of nitrogen, and no part of an organ contains less than 17 per cent. of nitrogen." Fat and water are components of the animal body which are destitute of nitrogen, but they are not considered as organized or living substances. For the development, nutrition, and renovation of all the living animal parts nitrogen is essential; it is therefore to be found in all the articles from which young animals derive their nourishment. It constitutes an element in the yolk of the egg, and is one of the elements in the caseine of milk. In the proteine compounds its average per-centage is over 15, in the gelatinous tissues about 18, in wheat dried in vacuo 2.3, in peas similarly dried 4.2, in dried potatoes 1.80, in cabbage dried ot 212°, 3.70, in carrots, also dried, 2.40, in turnips, in the same condition, 2.20. We have shown, in a former number, that it is the opinion of Liebig, as well as of other chemists, that it is only the *nitrogenized* elements which are capable of conversion into blood, and of forming organized tissues, and that the *non-nitrogenized* foods, although indispensable to the comfort and very existence of animals, are only so as being necessary to support the process of respiration—in other words, to maintain animal heat.

Some objections are brought forward by our author against this doctrine, but we shall not stop to consider them. The fact, now fully established, that the food of all animals, herbivorous and carnivorous, contains nitrogenized matters, identical in composition with the principal constituents of the blood, is of itself sufficient to render the hypothesis of Liebig highly probable. Fibrine, albumen and caseine, whether found in animal or vegetable compounds, are the same in composition, and they are amply abundant in the food of all classes of animals to supply the materials for the growth, nutrition, and reproduction of their parts. And if sufficient, what need for looking further for elements of nutrition—for supposing that the carbon and hydrogen of sugar, gum, starch and the oils, have any agency in the formation of blood? Still, it is possible that the other opinion may be true, that both sugar and fat are occasionally converted into nitrogenized animal substances. It has not been made out that nitrogen is never absorbed by the lungs, and admitting that it is, at times, imbibed with oxygen, its use may be to assimilate these matters to the living animal tissues. There is nothing impossible in the supposition, and although we do not deem it a probable one, we cannot doubt the power of the vital processes to effect such a change, any more than that, by the separation of a part of their nitrogen, they may convert the gelatinous tissues into proteine compounds. The arguments in favor of it will be found stated, with great clearness and force, by Per eira under the head of nitrogen. He insists, with much plausibility, that sugar takes some other part in digestion than merely to supply the blood with one of the elements of respiration. But we cannot pursue this subject further.

Phosphorus, a substance never seen except in the laboratory and shops, is yet an invariable constituent of many animal and vegetable substances, and an essential ingredient of fibrine and albumen, and of all the tissues into which they enter. It is contained in the nervous matter, in the brain, and in the bones, as well as in the spermatic fluid and the ovaria. It is a constituent of the flesh, the blood and the bones of animals which form our food. In the bones it exists as phosphoric acid, but in the soft parts the precise form it affects is not known. Fishes are especially rich in this matter, and it is also a much larger constituent of vegetable substances than was suspected before chemists had furnished us with the analysis of organic bodies. Unrefined sugar, garlic, potatoes, and the seeds of all the grasses, contain it, so that the herbivora derive it from this source in quantities greater than their systems require, and the excess occasionally gives rise to concrètions in the cœcum of the horse as large as the head of a child. Pereira mentions one of these *hippolithi* in his possession consisting of the ammoniacal phosphate of magnesia, which weighs between five and six pounds.

Phosphoric acid is described by chemists as possessing a singular pliancy of constitution, which seems to have adapted it, above all other mineral acids, to the wants of the animal economy. One of the salts of this acid, the metaphosphate of soda, by the agency of heat alone, exhibits a change of *nature*, without a change of *composition*, similar to what often occurs in organic compounds. This salt combines with water, which becomes attached to it as water of crystallization, and the salt is then a hydrate. Dried at 212°, it retains water, but not as a base, for on dissolving the salt again its constitution is found to be unaltered—it is still a metaphosphate. "But let this metaphosphate be heated to 300°, and *without losing anything*, *it changes completely*, and becomes a pyrophosphate, the water which was constitutional before, being now basic." This peculiarity of phosphoric acid fits it to make "one of the links by which mineral and organic compounds are connected."*

Sulphur is very widely disseminated among animal and vegetable bodies. In fibrine, albumen and caseine, as well as in the hair and bones, it is always present, and in some of these, probably, uncombined with oxygen. The vegetable kingdom is not deficient in this element. Celery, rice, hops, ginger, all the cruciferous tribe of plants, contain it. It is found in asafœtida, which is sometimes used as a condiment, and by some oriental tribes is considered as "*food for the gods.*"

The sulphuretted hydrogen, found in the alimentary canal, is supposed by our author to be often produced by the action of decomposing organic matters on the sulphates. "An eminent chemical philosopher tells me," he remarks, "that he is always much troubled with the evolution of this gas after the use of sulphate of magnesia." Every physician will be able to recall to his memory instances of this kind—cases in which towards a fatal termination, especially, sulphuric acid and the sulphates were decomposed, with the extrication of much sulphuretted hydrogen.

Iron has been long recognized as one of the constituents of organic beings, but it is a little remarkable that chemists are not yet agreed as to the precise state in which it exists in their tissues. Dr. Pereira observes,

"This metal is an essential constituent of the blood corpuscles, though, according to the recent researches of Scherer, it is neither essential to hæmatosin, nor necessary to the color of the blood. But the well-known beneficial influence of chalybeates in the disease called anæmia, in which the blood is found to contain a smaller quantity of iron than in a state of health, favors the notion that the proper color of this fluid is in some way connected with the amount of iron contained in it; for one of the most characteristic symptoms of this malady is an absence of the natural vermilion tint of the complexion."

---

* Graham's Elements of Chemistry, p. 256.

The American editor, Dr. Lee, adds an interesting note on this subject. He says:

"The physiological effects of a want of the usual proportion of iron in the blood globules, yet remain to be investigated. If Liebig's hypothesis be correct, then such a deficiency must cause the globules to lose their property of absorbing oxygen and of afterwards giving up this oxygen and carrying off the resulting carbonic acid, which would doubtless lead to important changes in the temperature and other vital phenomena of the body. The vital motions would go on, but the change of matter would be arrested; no lifeless compounds could consequently be separated, such as bile or urine, and the animal temperature would necessarily sink. The phenomena connected with aggravated cases of anæmia, in leucophlegmatic subjects, lend much plausibility to such doctrine."

Chlorine is found in the blood, and in several of the secretions. In the blood and the excretions it is combined with sodium, in the gastric juice it is united to hydrogen, and constitutes the old muriatic acid. It is constantly abstracted from the blood, in the production of the various fluids containing it, and must be continually restored to it again. This is done with the food, in the form of chloride of sodium. The embryo chick finds it in the egg, and the young mammal derives it from its mother's milk. Hydrochloric acid appears to be a necessary agent in the process of digestion, and, in fact, an artificial digestive liquor may be prepared by macerating the lining membrane of the fourth stomach of the calf in water, to which a few drops of this acid have been added. Beefsteak and albumen are gradually dissolved in this mixture, while they are not affected by either the acid or the infusion of the stomach alone. Liebig ascribes the action of the gastric fluid to the circumstance, that it is matter in a state of transformation, which state is communicated to the alimentary substances. A few drops of sour milk left in a pan will cause the fresh milk to ferment in a few hours. Here the milk, undergoing change, communicates the same tendency to the fresh body of the fluid—As yeast, which is matter undergoing transformation, imparts a fermentative tendency to the mass of flour in which it is mingled.

Dr. Pereira adduces several circumstances which are opposed to this theory of digestion. They are given in the following extract:

"The fact ascertained by Schwann, that the solvent principle of the digestive fluid can be precipitated from its neutral solution by acetate of lead, and be obtained again in an active state from the precipitate by means of sulphuretted hydrogen, is apparently inconsistent with Liebig's idea, that this principle is matter in a state of decomposition or transformation. Moreover, if the essential part of the gastric juice—that by which digestion is effected—be a mere transformation of the stomach, how is it that other parts of analogous structure and composition do not suffer the same transformation? I have tried to obtain a digestive liquor from the second stomach of the calf, and from the bladder, but in vain. How is it that this fancied transformation goes on, during life, only when solicited to do so by the presence of aliment or by mechanical irritation? Dr. Beaumont ascertained that pure gastric juice will keep for many months without becoming fetid: a fact scarcely explicable on the hypothesis that its activity depends on a principle in a state of decomposition. I find that while acidulated infusions of the second stomach of the calf, and of the bladder, soon become putrid and fetid, that of the fourth stomach remains remarkably free from unpleasant smell for several weeks. Lastly, I find, contrary to Liebig's statement, that a digestive liquor can be prepared from the fresh undried fourth stomach of a calf.

"I cannot agree with Liebig, that digestion is a process analogous to fermentation; that, in fact, it is nothing more than the transformation of food, effected by the contact of matter in a state of decomposition. If it were, a small quantity of gastric juice ought to be capable of effecting the digestion of an unlimited quantity of food. Now, the experiments of Dr. Beaumont on the natural gastric juice, and of Schwann on the artificial digestive liquor, prove that this is not the case. Both found that only a certain amount of food could be digested with a given quantity of gastric juice; and Dr. Beaumont observes, that "when the juice becomes saturated, it refuses to dissolve more; and if an excess of food have been taken, the residue remains in the stomach, or passes into the bowels in a crude state." Now, this fact is quite inconsistent with the fermentation theory."

We pass over the other chemical elements, calcium, magnesium, potassium, and fluorine, as, with the exception of the first, they are comparatively of less interest as constituents of our food.

Chapter II treats of *alimentary* principles, which Pereira divides into the following twelve classes; namely, *aqueous*, *mucilaginous*, *saccharine*, *amylaceous*, *ligneous*, *pectinaceous*, *acidulous*, *alcoholic*, *oily*, *proteinaceous*, *gelatinous*, and *saline*.

Water makes a large proportion of the human body. The blood contains about 80 per cent., and the flesh about 74 per cent. of it; so that we may assume, with our author, that of the entire human machine nearly three-fourths of its weight are water. Wasted as it is by the various vital processes, a continual supply of this fluid is indispensable to the life of animals, which have been known to subsist for many weeks without solid food, but cannot live many days without water. "In this point of view, it holds an intermediate rank between air and solid food, being less essential than the first, but more so than the last."

Water enters largely into the composition of most of our alimentary substances, in some forming true hydrates, and in others being present merely as accidental moisture. To the constitution of the first it is essential, and its removal by heat or chemical agency changes their nature. Sugar, starch, or albumen is decomposed when deprived of the water which is one of its elements; and, on the other hand, cane sugar or starch, may be converted into sugar of milk, or diabetic sugar by the addition of water. "So also the hydrochloric acid of the gastric juice and the soda of the blood and bile, are derived from common salt by the aid of water." Other cases, where a change of chemical constitution is effected by the instrumentality of water, are cited by Pereira.

In a dietetical point of view water may be regarded as to *quantity* and *quality*. As a remedy in fevers and acute inflammatory diseases, an almost unlimited use of aqueous fluids is admitted; but in other maladies we restrict the quantity, as in valvular diseases of the heart, when our object is to keep down the volume of the circulating fluid, or to prevent thinness of the blood, as in aneurism of any of the great vessels, in which our only hope is in the coagulation of fibrine within the sac, or when we seek to repress excessive secretion, as of urine in diabetes. Attention to the *quality* as well as the quantity of the water is also important, both in the cure and the prevention of disease. The purest is rain-water; that of wells and springs contains mineral ingredients derived from the strata through which it percolates. River water is a mixture of rain and spring water. It is deemed more wholesome than the water of springs. The health of Nashville, especially of the infant part of the population, is believed to have been improved by the introduction of river water into the city. Children are said to suffer less with bowel complaints than in former years. In the navy of Great Britain it has been ascertained that dysentery has become less prevalent, since care is exercised by the medical officers to have the men supplied with pure water.

The second alimentary principle of our author is *gum*, which exists almost universally in plants, "and appears," he remarks, "to hold the same position in the vegetable economy, that albumen does in the animal." Liebig denies that it possesses nutritive properties, and alleges that its only use as an article of diet, is to furnish fuel for the oxygen consumed in respiration. Prout holds that it is not converted into sugar in the digestive process, and in view of this property, Pereira suggests that it might be advantageously substituted for sugar and amylaceous substances in diabetes. It is found an indigestible article of diet by dyspeptics.

The saccharine principle is a very widely distributed and very important one. "Sugar," says Graham, "seems to be the agent, by means of which plants as well as animals develope the heat they require. The fecundation of plants is always accompanied by heat, the flowers respiring and producing carbonic acid. They must therefore consume carbon, and accordingly we find that the sugar in the stems of the sugar-cane has entirely disappeared after the flowering and fructification are completed."* The same remark is applicable to the indian corn, the stalk of which is found destitute of sugar after the maturing of the grain. The essential purpose of sugar, according to Liebig, is the development of vital heat, and hence it is an ingredient of the food of all young animals which derive nourishment from the mother, as it is also in that of the embryo plant before its rootlets have permeated the soil. If this principle be supplied to an animal in excess, that portion which is not consumed in respiration is deposited in its system in the shape of fat. It is not, according to his view, one of the blood-making compounds.

Pereira esteems sugar a healthy article of diet, and supposes that the erroneous notion of its being injurious to the teeth, "has been propagated by frugal housewives in order to deter children from indulging in an expensive luxury." He cites the case of Henry, Duke of Beaufort, who died of fever at the advanced age of 70, having eaten nearly a pound of sugar daily for 40 years. "He was never troubled with cough, his teeth were firm, and all his viscera were found, after death, quite sound." Some of the circumstances which forbid the use of sugar as a diet, are indicated in the following passage:

"In diabetes, the power of assimilating saccharine matter is in a great measure, if not wholly, lost; and hence, therefore, the dietetical employment of sugar and sweet foods, in this malady, is highly improper. In the oxalate of lime diathesis, likewise, these foods are objectionable. "I have seen repeated cases," says Dr. Prout, "in which the too free use, or rather abuse, of sugar, has given occasion to the oxalic acid form of dyspepsia; and sooner or later, under favorable circumstances, to the formation of an oxalate of lime calculus." In the phosphatic diathesis, the copious use of unrefined sugar is objectionable, on account of the lime contained in it."

---

Op. cit. p. 689.

Starch, the *amylaceous* principle of our author, is generally distributed in the vegetable kingdom, existing in endogenous and exogenous plants, and being found in roots, stems, tubercles, fruits and seeds. Iceland moss contains 44 parts in the hundred of this principle; tapioca from 7 to 13, potatoes from 9 to 24, arrow-root from 12 to 26, wheat flour 56 to 74, maize nearly 81, and rice more than 85 per cent. To render amylaceous matter digestible in the human stomach it must be cooked, as the heat of that organ, according to Raspail, is not sufficient to burst the grains of the feculent mass. In graminivorous animals and birds the power of the digestive apparatus is greater, but many experiments show that a great advantage results from boiling the farinaceous substances given them for food. By digestion starch is converted into gum and sugar, the latter being probably absorbed, and entering the circulation, according to Liebig, to form an element of respiration. Prout, on the contrary, regards starch as a mild, slightly nutritious, easily digestible article of food, of which man and the higher animals can partake in much larger quantities than sugar, and for an unlimited period. Animals grow fat upon it, but Liebig alleges that it neither developes their organs, nor increases their strength. In the form of sago, arrow-root, tapioca, Iceland moss, or water-gruel, it constitutes an unstimulating diet for persons affcted with morbid sensibility of the primæ viæ, as well as for the subjects of fever and inflammatory diseases.

Our author questions the claim of the *ligneous* principle to a place among nutritious articles. It rests on the statement of Autenrieth, that wood reduced to powder, repeatedly subjected to the heat of an oven, and then ground in the manner of corn, yields a flour which may be made into a perfectly uniform bread. But Pereira supposes that the nutriment found in such bread comes from the starch, which, in the autumn, after the formation of wood has ceased, is diffused through every part of the plant by the autumnal sap. Heyer, by means of a microscope, detected starch thus deposited in the bodies of trees.

*Jelly* is the *pectinaceous* alimentary principle of Pereira, and abounds in fruits. He says:

"According to Fremy, unripe fruits contain a very small portion only of pectine; but when the fruit becomes ripe, pectine is formed by the action of the vegetable acids of the fruit on a pulpy matter. These acids are contained in cells, from which they do not escape until the period of ripening, when the cells are transparent, distended, and permeable. By subjecting fruit to heat the cells burst and allow the acid to escape, and in this way the formation of pectine is promoted."

Pereira believes "vegetable jelly to be slightly nutritive, and readily digestible." Containing no nitrogen, Liebig places it with sugar and starch, among the elements of respiration.

The acidulous elementary principle is widely distributed among vegetables, and as an article of diet has been used in all periods, and among all nations, except those inhabiting the coldest latitudes. To such it would not appear to be a necessary article of food. They enjoy good health upon an unmixed animal diet; but in warm countries it is undoubtedly necessary to the preservation of health. Its efficacy in preventing scurvy is well known, and in fevers, water sharpened with the vegetable acids makes a beverage which is at the same time grateful and medicinal. But the use of these acids, especially that of vinegar, may be carried too far. Our author cites the following case as showing that the practice, not uncommon among young ladies, of employing it to diminish obesity, is attended with danger:

"A few years ago, a young lady, in easy circumstances, enjoyed good health; she was very plump, had a good appetite, and a complexion blooming with roses and lilies. She began to look upon her plumpness with suspicion; for her mother was very fat, and she was afraid of becoming like her. Accordingly, she consulted a woman, who advised her to drink a small glass of vinegar daily; the young lady followed her advice, and her plumpness diminished. She was delighted with the success of the remedy, and continued it for more than a month. She began to have a cough; but it was dry at its commencement, and was considered as a slight cold, which would go off. Meantime, from dry it became moist; a slow fever came on, and a difficulty of breathing; her body became lean, and wasted away; night-sweats, swelling of the feet and of the legs succeeded, and a diarrhœa terminated her life. On examination, all the lobes of the lungs were found filled with tubercles, and somewhat resembling a bunch of grapes."

*Alcohol* is scarcely entitled to a place among alimentary principles. Of all truly nutritious qualities it is admitted to be destitute, and even as an element of respiration its pretensions are questionable. Its influence in promoting animal heat may well be doubted, since it is proved by experience that, other things being equal, men indulging in alcoholic drinks perish sooner from cold, than those who entirely abstain from them. On no ground can the habitual use of such drinks by healthy people be justified, and the profession is called upon by every solemn obligation, to discountenance a practice fraught with the worst consequences to individuals and society.

The *oily* alimentary principle is derived from both the organic kingdoms. It is found in the shape of fixed and volatile oils. Bile, according to Beaumont, accelerates the chymification of oils, and hence small quantities of that fluid are found "mixed with the gastric juices where the use of fat or oily food has been persevered in for some time." In many dyspeptics fat does not become properly chymified, but floats on the contents of the stomach, becoming highly rancid, and exciting heartburn, eructations, nausea, and sometimes vomiting. Butter is less apt to disagree with such patients than any other of the fixed oils, but in many persons of bad digestion we have found broiled bacon to agree better with the stomach than any other article of food, and Dr. Lee makes the same remark concerning children laboring under a chronic form of cholera infantum. This principle is not nutritious, according to the views of Liebig, and the following facts are stated by our author on that subject:

"In the report made to the French Academy of Sciences, in the name of the Gelatine Commission, it is stated that animals fed on fatty substances (fresh butter, lard, and the fat which surrounds the bullock's heart) refuse, after some time, to take this food, and ultimately die of inanition. During life, they exhaled a strong fatty odor, and, though dying of inanition, were in a remarkable state of *embonpoint.* On a post-mortem examination, all the tissues and organs were found infiltrated with fat, and the liver was in the state called by anatomists *fatty.*"

Fibrine, albumen, and caseine contain, as their basis, a substance to which Mulder has affixed the term *proteine;* these constitute a group of alimentary principles termed by Pereira the *proteinaceous.* They are "the plastic elements of nutrition," according to Liebig, and are derived by animals from vegetable matters, in which alone they are produced. But it is remarkable that, while they are the true nutritious elements, not one of them singly is capable of supporting animal life. Magendie fed dogs on fibrine until they died of inanition, and on post-mortem examination it was found, that the blood had almost entirely disappeared, all that was left affording only a few grains of fibrine. We have referred, on other occasions, to the experiments of Tiedemann and Gmelin on geese, in which albumen was fed to these animals with similar results. Still, as Liebig holds, albumen must be regarded as "the true starting point of all the animal tissues." Out of the white and yolk of the egg, the blood of the young bird, the muscles, feathers, nerves, and blood vessels are fabricated, aided only by atmospherical oxygen, and the oil and iron mingled with the albumen.

The vegetable proteinaceous principles are identical, according to the analysis of Mulder and Scherer with the fibrine, albumen, and caseine derived from animals. By the animal processes, they are impressed with the peculiar shape and characteristics which they assume as blood, muscular fibre, and the various tissues of the animal body.

" 'How beautifully and admirably simple,' says Liebig, 'appears the process of nutrition in animals, the formation of their organs in which vitality chiefly resides! Those vegetable principles which, in animals, are used to form blood, contain the chief constituents of blood, fibrine, and albumen, ready formed, as far as regards their composition. All plants, besides, contain a certain quantity of iron, which reappears in the coloring matter of the blood. Vegetable fibrine and animal fibrine, vegetable albumen and animal albumen, hardly differ, even in form; if these principles be wanting in the food, the nutrition of the animal is arrested; and when they are present, the graminivorous animal obtains in its food the very same principles on the presence of which the nutrition of the carnivora entirely depends. Vegetables produce in their organism the blood of all animals, for the carnivora, in consuming the blood and flesh of the graminivora, consume, strictly speaking, only the vegetable principles which have served for the nutrition of the latter. Vegetable fibrine and albumen take the same form in the stomach of the graminivorous animal as animal fibrine and albumen do in that of the carnivorous animal.' "

The foregoing is quoted without comment by Pereira. Dumas has recently expressed himself in language very similar to that of Liebig. Differing as these eminent chemists do on several points of chemico-physiology, the concurrence of their views in regard to animal digestion heightens the probability of their theory. Dumas thus speaks of that process:

"Digestion is a simple function of absorption. The soluble matters pass into the blood, for the most part unaltered; the insoluble matters arrive in the chyle sufficiently divided to be aspired by the orifices of the chyliferous vessels. Digestion has evidently for an object to restore to the blood, a matter proper to furnish for our respiration the ten or fifteen grammes of carbon or the equivalent of hydrogen, which every individual burns per hour, and also to provide the gramme of nitrogen, which is exhaled every hour, in part by the lungs and skin, as well as by the urine. Thus, amylaceous matters are converted into gum and sugar; the saccharine matters formed are absorbed. The fat matters are divided, form an emulsion, and so pass into the vessels, to form afterwards deposits, which the blood takes up and burns, when they are required. The neutral azotised matters, the fibrine, albumen, and caseine, first dissolved, then precipitated, pass into the chyle highly divided or dissolved anew.

"An animal, therefore, receives and assimilates almost untouched, the neutral azotised matters which he finds ready formed in the animals or plants upon which he lives; he receives oily substances which come from the same sources, and also amylaceous and saccharine substances of the same origin. These three orders of matters, of which the origin is always traceable to the plant, divide themselves into products admitting of assimilation; into fibrine, albumen, caseine, and oily bodies, which serve to increase or renew the organs; and into combustible products, sugar and the oily bodies, which are consumed in respiration. An animal thus assimilates or destroys ready formed organic matters; it creates nothing."

We here close, for the present, our notice of this interesting work. The part which treats of compound aliments, amounting to more than half of the volume, will probably form the subject of a future article. If, in the estimation of some of our readers, we have dwelt with tedious minuteness upon certain chemical questions, our apology is, that they are topics which are exciting unusual attention in the medical world, at the present day, and are every where the subjects of animated discussion. Our next article, however, will be occupied with matters more practical in their nature. Y.

